# Kidney tubule injury is associated with sodium avidity and diuretic responsiveness in acute heart failure

**DOI:** 10.1093/eschf/xvag079

**Published:** 2026-03-13

**Authors:** Yu Horiuchi, Stephen Duff, Dirk J van Veldhuisen, Michelle M Estrella, Michael G Shlipak, Yoshimitsu Takaoka, Patrick T Murray, Joachim H Ix, Nicholas Wettersten

**Affiliations:** Division of Cardiology, Mitsui Memorial Hospital, Tokyo, Japan; School of Medicine, University College Dublin, Dublin, Ireland; Department of Cardiology, University Medical Center Groningen, University of Groningen, Groningen, the Netherlands; Nephrology Section, San Francisco Veterans Affairs Healthcare System, San Francisco, CA, USA; Kidney Health Research Collaborative, Department of Medicine, SanFrancisco VA Healthcare System, San Francisco, CA, USA; Kidney Health Research Collaborative, Department of Medicine, SanFrancisco VA Healthcare System, San Francisco, CA, USA; Division of Cardiovascular Medicine, University of California, San Diego, La Jolla, CA, USA; School of Medicine, University College Dublin, Dublin, Ireland; Division of Nephrology-Hypertension, Department of Medicine, University of California San Diego, and Veterans Affairs San Diego Healthcare System, San Diego, CA, USA; Division of Cardiovascular Medicine, University of California, San Diego, La Jolla, CA, USA; Division of Cardiovascular Medicine, San Diego Veterans Affairs Medical Center, Cardiology 4 West, Room 4022 (111A), 3350 La Jolla Village Drive, San Diego, CA 92161, USA

**Keywords:** Acute heart failure, Natriuresis, Kidney tubule biomarkers, Diuretic resistance

## Abstract

**Introduction:**

Greater sodium avidity in acute heart failure (AHF) is associated with worse outcomes, but whether kidney tubule injury is associated with sodium avidity and impaired diuretic responsiveness remains underexplored.

**Methods:**

We evaluated 339 participants from the ROSE-AHF trial, which enrolled patients hospitalized for AHF with kidney dysfunction and randomized them to the dopamine, nesiritide, or placebo group. Urinary kidney injury molecule-1 (KIM-1), *N*-acetyl-β-D-glucosaminidase (NAG), and neutrophil gelatinase-associated lipocalin (NGAL) were measured at enrolment. Associations between these biomarkers and urinary sodium (uNa) concentration at baseline, fractional excretion of sodium (FeNa), as well as total uNa output and urine output over 72-h were assessed using multivariable regression models.

**Results:**

Higher KIM-1 and NAG values at baseline were associated with lower uNa concentration at baseline [−6.1% (−8.5%, −3.7%), *P* < 0.001 and −5.9% (−9.2%, −2.6%), *P* & .001, respectively, per two-fold increase in each biomarker]. Higher baseline KIM-1 and NAG were also associated with lower FeNa [−6.1% (−8.5%, −3.6%), *P* < .001 and −5.2% (−8.6%, −3.6%), *P* = 0 .001, respectively, per two-fold increase in each biomarker]. Higher baseline KIM-1 was associated with lower total uNa excretion over 72-h [−3.6% (−6.8%, −0.2%), *P* = 0.037 per two-fold increase]. None of the biomarkers were associated with urine output over 72-h.

**Conclusion:**

Kidney tubular injury, as assessed by urine KIM-1 and NAG, is associated with greater sodium avidity and higher KIM-1 is associated with impaired diuretic responsiveness in AHF.

## Graphical Abstract

**Graphical Abstract:**
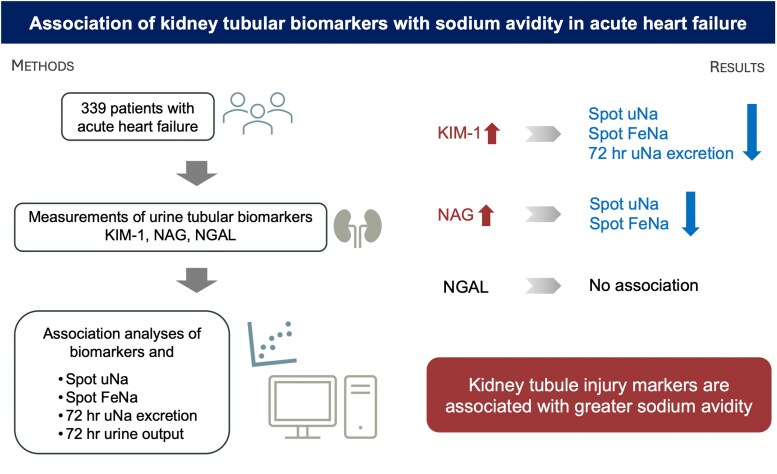
Associations between urinary kidney tubular injury biomarkers and measures of diuretic responsiveness and renal sodium avidity (spot urine sodium, spot fractional excretion of sodium, total urine sodium, and 72-h urine output) were assessed in ROSE-AHF. Higher levels of KIM-1 and NAG were associated with reduced urinary sodium excretion, whereas NGAL was not associated with any sodium measures. None of the biomarkers were associated with urine output. FeNa, fractional excretion of sodium; KIM-1, kidney injury molecule-1; NAG, *N*-acetyl-β-D-glucosaminidase; NGAL, neutrophil gelatinase-associated lipocalin; ROSE-AHF, Renal Optimization Strategies Evaluation Acute Heart Failure; uNa, urine sodium.

## Introduction

Acute heart failure (AHF) is a severe condition characterized by dyspnoea and organ dysfunction, often resulting from fluid overload from greater sodium avidity in the kidney.^1^ Diuretics serve as a primary treatment for AHF by enhancing sodium excretion into the urine, thereby alleviating volume overload. Impaired urine sodium (uNa) excretion during diuretic therapy is associated with poor clinical outcomes, and the assessment of uNa concentration has been advocated as a measure of diuretic responsiveness because sodium output directly indicates renal responsiveness to diuretic therapy.^2–4^ However, the response to diuretic therapy is often impaired during decompensations of HF, resulting in insufficient fluid removal, residual congestion, worsening HF, and a greater risk of death and HF readmission.^5,6^

Among the mechanisms underlying impaired diuretic response, kidney tubular adaptations and dysfunction may be particularly important because of the tubule’s role in the pharmacology of loop diuretics and sodium reabsorption.^7,8^ Loop diuretics, the cornerstone of diuretic therapy, are secreted from blood by the proximal tubule cells into the lumen, where they inhibit the Na^+^-K^+^-2Cl^−^ cotransporter on the luminal membrane of epithelial cells in the thick ascending limb. Other diuretics, like acetazolamide, sodium-glucose co-transport 2 inhibitors, and thiazides, also rely on intact tubular function to exert their effects.^9–11^ Thus, kidney tubular dysfunction from pathologic processes such as tubular injury can compromise the response to and efficacy of diuretics, leading to reduced sodium excretion and diminished therapeutic effectiveness.^12^ Additionally, tubular adaptations in the proximal or distal tubule outside of the Loop of Henle may have enhanced sodium avidity reducing the natriuretic effects of loop diuretics.^13^ Despite its potential significance, the relationship between tubular injury and diuretic resistance in AHF has not been thoroughly investigated. Finding biomarkers of tubular injury that identify individuals who are more likely to have an impaired response to diuretic therapy or greater sodium avidity may enable the initiation of more aggressive diuretic strategies immediately upon admission, potentially accelerating congestion relief, reducing the risk of developing worsening HF, and improving patient outcomes.

To explore this issue, we evaluated whether urine biomarkers of kidney tubule injury were associated with sodium excretion during hospitalization for AHF in the multicentre, randomized controlled Renal Optimization Strategies Evaluation (ROSE-AHF) trial.^14^ We hypothesized that greater kidney tubule injury would be associated with greater sodium avidity and reduced diuretic responsiveness.

## Methods

### Patient population

The rationale and methodology of the ROSE-AHF trial have been described previously.^14^ Briefly, ROSE-AHF enrolled 360 patients within 24 h of admission for AHF with kidney dysfunction, defined as an estimated glomerular filtration rate (eGFR) between 15 and 60 ml/min/1.73 m^2^. Participants were randomized 1:1 to either dopamine or nesiritide, then randomized 2:1 to therapy or placebo with placebo arms combined at the end effectively having 1:1:1 randomization to dopamine, nesiritide or placebo to determine whether either active therapy led to greater diuresis at 72-h or reduced the risk of acute kidney injury defined by an increase in cystatin C at 72-h. All patients received open-label, intravenous loop diuretic treatment with a recommended total daily dose equal to 2.5 times the total daily oral outpatient furosemide (or equivalent) dose before admission up to a maximum of 600 mg/day. Patients naive to outpatient loop diuretics received intravenous furosemide at 80 mg/day. Half the total daily diuretic dose was administered as a bolus twice daily for at least 24 h. Use of other medications and diuretic dosing after 24 h were at the discretion of the clinician.

Of the original 360 participants enrolled, 21 were missing kidney tubule biomarker measurements at the time of enrolment, leaving 339 individuals for evaluating the association of biomarkers with uNa at baseline. Of these 339 individuals, 266 had complete data on urine sodium output over the 72-h of follow-up. There were no significant differences between the 266 individuals included in the analyses over 72-h and the 73 individuals excluded (Supplementary Table S1). The ROSE-AHF trial was conducted under the support of the Heart Failure Clinical Trials Network (HF-CTN), sponsored by the National Heart, Lung, and Blood Institute. The study protocol was approved by the institutional review boards of all participating sites, and written informed consent was obtained from all participants prior to randomization. The data and research materials used in this analysis were acquired directly from the National Heart, Lung, and Blood Institute's BioLINCC repository.

### Measurement of biomarkers

As previously reported, plasma creatinine, cystatin C, and N-terminal pro-B-type natriuretic peptide (NT-proBNP) levels were measured at a central laboratory (HF-CTN Core Biomarker Laboratory, University of Vermont).^15^ After study enrolment and before initiation of randomized therapy, a spot urine specimen was collected for uNa concentration and urine biomarker measurements. This specimen was collected randomly and not timed to previous loop diuretic administration. Over the subsequent 72-h, all urine was collected in 24-h intervals for measurement of total urine output and uNa concentration of urine. From these 24-h collections, the uNa concentration was multiplied by total urine volume to calculate the total amount of uNa excreted. These 24-h intervals were summed for total urine and uNa output over 72-h. Additionally, spot urine specimens were collected at 24-h intervals from time of enrolment, and these specimen collections were not timed to loop diuretic administration.

For urine tubular injury biomarkers, urine samples were processed using microbeads conjugated with NGAL (Enzo Lifesciences) and KIM-1 (R&D Systems) antibodies and analysed with the Bio-Plex 200 system (Bio-Rad). Urinary NAG levels were determined using a commercially available NAG assay kit following the manufacturer’s protocol (Roche Diagnostics). The intra- and inter-assay coefficients of variation reported for urine-based assays are <5% and <10% for KIM-1, <5% and <5% for NAG, and <5% and <10% for NGAL, respectively.^16–18^

### Outcomes

Our primary outcome was uNa concentration (*n* = 339) at the time of enrolment. Our secondary outcomes included the fractional excretion of sodium (FeNa) at time of enrolment (*n* = 339), total uNa output over 72-h (*n* = 266), and total urine output over 72-h (*n* = 266).

### Statistical analysis

Baseline characteristics are presented as means (standard deviations), medians (first and third quartiles), or counts (percentages). Estimated glomerular filtration rate was calculated using the Modification of Diet in Renal Disease equation as done originally in ROSE-AHF.^14,19^ Baseline characteristics were evaluated between the dopamine, nesiritide, and placebo groups with the chi-square test, ANOVA, and Kruskal–Wallis, as appropriate. Correlations between urine tubule biomarkers, creatinine, and eGFR were evaluated using Spearman’s rank correlation.

Values of biomarkers were right-skewed, and thus, log base-2 transformed to achieve a more normal distribution and to allow associations to be interpreted as ‘per two-fold higher’ of the biomarker in models. While distributions of uNa, total uNa output over 72-h, and total urine output over 72-h were relatively normal, FeNa was right-skewed. Thus, we log base-2 transformed all the dependent variables such that all associations can be interpreted similarly as the percent change in the dependent variable per two-fold higher of the biomarker value.

The association between urine tubule biomarkers and outcomes was assessed in linear regression models. Model 1 evaluated the association of each biomarker adjusted for 1/urine creatinine (uCr) to control for urine dilution. For uNa and FeNa at baseline, Model 2 adjusted for 1/uCr, serum creatinine, blood urea nitrogen, hypertension, atrial fibrillation, diastolic blood pressure, outpatient diuretic dose, and NT-proBNP levels. These variables were chosen as they had previously been identified as predictors of diuretic efficiency from an analysis of HF-CTN trials which included ROSE-AHF.^20^ For total uNa output and urine output over 72-h, Model 2 was adjusted for the same variables as Model 2 for baseline outcomes, as well as the total loop diuretic dose administered over 72-h, use of thiazide diuretics during each 24-h period within the 72-h follow-up, and a composite congestion score during each 24-h period within the 72-h follow-up. The composite congestion score consisted of daily assessments of jugular venous pressure, orthopnoea, and peripheral oedema (Supplementary Table S2) like prior congestion scores.^5,21^ Because some covariates were missing in up to 10% of data, we performed multiple imputations by chained equations with a total of 10 imputations using all the variables from the fully adjusted model. Estimates were combined using Rubin’s rule to account for variability in the imputation procedure.^22^

Biomarkers were evaluated as continuous variables and by quartiles to assess their functional form. To explore potential non-linear associations between biomarkers and dependent variables, we evaluated each biomarker with restricted cubic splines with three to five knots based on the optimal Akaike Information Criterion. We tested for significance of non-linearity and displayed the percent change in dependent variable for two-fold change in biomarker referenced to the median biomarker value. We evaluated biomarkers individually in models and with all three combined in a model. We evaluated for collinearity between biomarkers by assessing the variance inflation factor. We evaluated for effect modification by left ventricular ejection fraction (LVEF) by testing the interaction between biomarkers and LVEF with patients dichotomized as LVEF <50% or ≥50%. We also evaluated for effect modification by eGFR by testing the interaction between biomarkers and eGFR with eGFR categorized as ≥45 ml/min/1.73 m^2^, 30–<45 ml/min/1.73 m^2^, or <30 ml/min/1.73 m^2^. For measurements of total uNa and urine volume over 72-h, effect modification by the treatment arm was assessed by testing for an interaction between biomarkers and the treatment arm.

All statistical tests were assessed with a two-sided *P*-value <.05, indicating significance. Analyses were performed using R version 4.2.2 (http://www.r-project.org).

## Results

### Study characteristics

The mean age of the study population was 70 ± 12 years, and 73% were men (*Table 1*). Most participants had a history of hypertension (84%), and the majority had ischaemic cardiomyopathy (58%). The mean LVEF was 37 ± 17%. The median outpatient furosemide dose was 80 [60, 160] mg. The mean value of eGFR was 45 ± 15 ml/min/1.73m^2^, and the median NT-proBNP was 4972 [2330, 10120] pg/ml. There were no differences in baseline characteristics by treatment arm. Baseline uNa concentration, FeNa, total uNa excretion at 72-h, and total urine output over 72-h were not different between treatment arms.

**Table 1:** Baseline characteristics of the study population

	Overall	Placebo	Dopamine	Nesiritide	*P*-value
	*n* = 339	*n* = 112	*n* = 116	*n* = 111	
Age, years, mean (SD)	70 (12)	70 (12)	71 (11)	69 (13)	0.422
Male, *n* (%)	248 (73)	82 (73)	80 (69)	86 (77)	
Race, *n* (%)					
White	255 (75)	86 (77)	85 (73)	84 (76)	0.550
Black	70 (21)	21 (19)	26 (22)	23 (21)	
Other	12 (4)	3 (3)	5 (4)	4 (4)	
Unknown	2 (1)	2 (2)	0	0	
BMI, kg/m^2^, mean (SD)	32.4 (8.0)	33.0 (8.1)	32.1 (8.8)	32.1 (7.0)	0.616
Years of heart failure, mean (SD)	6.3 (6.0)	5.8 (5.4)	6.9 (7.5)	6.1 (4.8)	0.399
LVEF, %, mean (SD)	37 (17)	35 (17)	37 (16)	38 (18)	0.533
Systolic blood pressure, mmHg, mean (SD)	118 (19)	117 (19)	118 (19)	117 (19)	0.885
Diastolic blood pressure, mmHg, mean (SD)	66 (11)	67 (12)	66 (11)	66 (11)	0.581
Ischaemic cardiomyopathy, *n* (%)	197 (58)	69 (62)	68 (59)	60 (54)	0.516
History of myocardial infarction, *n* (%)	120 (35)	41 (37)	42 (36)	37 (33)	0.856
Hypertension, *n* (%)	283 (84)	95 (85)	95 (82)	93 (84)	.833
Atrial fibrillation, *n* (%)	204 (60)	68 (61)	75 (65)	61 (55)	.325
COPD, *n* (%)	88 (26)	32 (29)	30 (26)	26 (23)	.681
Diabetes, *n* (%)	189 (56)	63 (56)	68 (59)	58 (52)	.622
Hyperlipidemia, *n* (%)	266 (79)	93 (83)	87 (75)	86 (77)	.321
ACEi or ARB, *n* (%)	166 (49)	57 (51)	50 (43)	59 (53)	.281
Beta-blocker, *n* (%)	282 (83)	96 (86)	94 (81)	92 (83)	.637
MRA, *n* (%)	99 (29)	31 (28)	31 (27)	37 (33)	.500
Outpatient loop dose, median [IQR]	80 [60, 160]	120 [60, 240]	80 [60, 160]	80 [80, 160]	.311
BUN, mg/dl, mean (SD)	43 (22)	42 (18)	42 (24)	43 (23)	.924
Creatinine, mg/dl, mean (SD)	1.73 (0.54)	1.75 (0.53)	1.71 (0.59)	1.73 (0.51)	.886
Cystatin C, mg/dl, mean (SD)	1.82 (0.57)	1.83 (0.54)	1.82 (0.65)	1.80 (0.49)	.925
eGFR, ml/min/1.73 m^2^, mean (SD)	45 (15)	44 (14)	46 (17)	45 (13)	.545
eGFR Categories					
eGFR <30 ml/min/1.73 m^2^, *n* (%)	69 (20)	26 (23)	26 (22)	17 (15)	.309
eGFR 30-<45 ml/min/1.73 m^2^, *n* (%)	125 (37)	41 (37)	37 (32)	47 (42)	.544
eGFR ≥45 ml/min/1.73 m^2^, *n* (%)	145 (43)	45 (40)	53 (46)	47 (42)	.699
NT-proBNP, pg/ml, median [IQR]	4972 [2330, 10120]	5054 [2319, 9808]	5733 [2940, 11893]	4502 [1859, 9114]	.163
KIM-1, pg/ml, median [IQR]	373 [145, 965]	384 [124, 1004]	351 [161, 770]	387 [128, 1008]	.834
NAG, mU/ml, median [IQR]	3.5 [1.7, 6.2]	3.2 [1.8, 5.8]	3.5 [1.9, 5.8]	3.9 [1.7, 7.9]	.657
NGAL, ng/ml, median [IQR])	26.2 [6.0, 123.4]	32.7 [9.8, 123.1]	26.2 [4.7, 117.2]	25.5 [5.4, 123.6]	.647
UACR, mg/g, median [IQR]	17 [4.1, 68]	55 [12, 186]	40 [15, 202]	38 [11, 106]	.479
Baseline urine sodium, mmol/l, mean (SD)	57 (28)	60 (29)	57 (28)	55 (28)	.383
Baseline FeNa, %, median [IQR]	2.00 [0.8, 4.0]	2.14 [1.0, 4.3]	2.0 [0.7, 3.92]	1.79 [0.9, 3.6]	.489
Total urine sodium over 72-h, mmol, mmol, mean (SD)	522 (288)	549 (313)	500 (284)	514 (263)	.494
Total urine volume over 72-h, ml, mean (SD)	8565 (2977)	8523 (2930)	8478 (3100)	8701 (2931)	.876

ACEi, angiotensin-converting enzyme inhibitor; ARB, angiotensin receptor blocker; AF, atrial fibrillation; BMI, body mass index; BUN, blood urea nitrogen; COPD, chronic obstructive pulmonary disease; eGFR, estimated glomerular filtration rate; FeNa, fractional excretion of sodium; KIM-1, kidney injury molecule-1; LVEF, left ventricular ejection fraction; MRA, mineralocorticoid receptor antagonist; NGAL, neutrophil gelatinase-associated lipocalin; NAG, *N*-acetyl-β-D-glucosaminidase; NT-proBNP, N-terminal pro B-type natriuretic peptide; UACR, urine albumin-to-creatinine ratio.

The correlations among glomerular filtration markers and markers of tubular injury are shown in Supplementary Figure S1. Each marker exhibited a weak to moderate correlation with the others (|*r*| ≤ 0.21). When comparing differences in treatments and treatment responses by quartiles of biomarkers, there was no difference in median loop diuretic dose, thiazide use, congestion status, or total urine output at 72-h across quartiles of KIM-1, while uNa concentration, FeNa, and total uNa output were significantly lower with higher quartiles of KIM-1 (*Table 2*). Trends were similar for NAG except median diuretic dose was higher with higher quartiles of NAG, while there was no difference in total uNa output (*Table 2*). Conversely, there were no differences in uNa concentration, FeNa or total uNa output across quartiles of NGAL, but there was lower total urine output and greater congestion status with higher quartiles (*Table 2*).

**Table 2:** Treatments, treatment outcomes and congestion statuses across quartiles of KIM-1

KIM-1 (pg/ml)	Quartile 1 (5.0, 144.6)	Quartile 2 (144.6, 372.9)	Quartile 3 (372.9, 965.2)	Quartile 4 (965.2, 15553.9)	*P*-trend
Loop naïve, *n* (%)	5 (6%)	4 (5%)	2 (2%)	8 (9%)	.457
uNa concentration, mmol/l, mean (SD)	66.1 (26.1)	61.8 (29.2)	52.6 (23.6)	49.0 (29.8)	<.001
FeNa, %, median [IQR]	3.0 [1.6, 4.8]	2.0 [0.9, 3.5]	1.8 [0.7, 3.8]	1.0 [0.5, 2.9]	<.001
Total uNa over 72-h, mmol, mean (SD)^a^	593 (301)	500 (225)	514 (300)	481 (304)	.036
Total urine output over 72-h, ml, mean (SD)^a^	8981 (2713)	8075 (2924)	8657 (3028)	8495 (3202)	.561
Total loop diuretic dose before enrolment, mg, median [IQR]	80 [40, 145]	80 [40, 125]	80 [40, 160]	80 [50, 150]	.773
Total loop diuretic dose over 72-hours, mg, median [IQR]^a^	537 [320, 780]	690 [400, 900]	590 [345, 729]	580 [350, 848]	.850
Thiazide used over 72-h, *n* (%)^a^	11 (17%)	14 (23%)	15 (23%)	19 (26%)	.174
Congestion score at enrolment, mean (SD)	9.3 (1.7)	9.0 (2.0)	9.3 (2.0)	9.3 (2.1)	.802
Congestion score at 24 h, mean (SD)^a^	5.9 (2.0)	5.8 (2.0)	5.9 (2.1)	6.3 (2.3)	.342
Congestion score at 48 h, mean (SD)^a^	4.8 (1.8)	5.2 (2.2)	5.1 (2.2)	5.7 (2.2)	.022
Congestion score at 72-h, mean (SD)^a^	4.3 (1.9)	4.2 (2.0)	4.1 (2.2)	4.8 (2.2)	.174

NAG (mU/ml)	Quartile 1 (0.02, 1.74)	Quartile 2 (1.74, 3.51)	Quartile 3 (3.51, 6.20)	Quartile 4 (6.20, 200.69)	*P*-trend
Loop naive, *n* (%)	8 (9%)	3 (4%)	3 (4%)	5 (6%)	.346
uNa concentration, mmol/l, mean (SD)	66.4 (26.3)	59.7 (28.3)	56.7 (29.5)	46.7 (25.6)	<.001
FeNa, %, median [IQR]	3.6 [2.1, 5.6]	2.3 [1.2, 4.0]	1.8 [0.8, 3.7]	0.8 [0.4, 1.9]	<.001
Total uNa over 72-h, mmol, mean (SD)^a^	542 (270)	549 (295)	505 (251)	493 (333)	.226
Total urine output over 72-h, ml, mean (SD)^a^	8483 (2726)	8935 (2938)	8470 (2777)	8388 (3454)	.660
Total loop diuretic dose before enrolment, mg, median [IQR]	80 [40, 160]	80 [40, 160]	80 [40, 140]	80 [50, 140]	.590
Total loop diuretic dose over 72-hours, mg, median [IQR]^a^	480 [240, 681]	590 [400, 944]	590 [435, 897]	600 [392, 910]	.017
Thiazide used over 72-h, *n* (%)^a^	13 (19%)	17 (27%)	15 (23%)	14 (21%)	.856
Congestion score at enrolment, mean (SD)	9.4 (2.0)	9.4 (1.8)	8.7 (2.0)	9.4 (2.1)	.366
Congestion score at 24 h, mean (SD)^a^	5.8 (2.0)	5.9 (2.2)	5.7 (2.0)	6.5 (2.3)	.154
Congestion score at 48 h, mean (SD)^a^	5.1 (2.0)	5.1 (2.4)	4.8 (1.8)	5.8 (2.2)	.103
Congestion score at 72-h, mean (SD)^a^	4.2 (1.9)	4.2 (2.3)	3.9 (1.7)	5.2 (2.2)	.020

NGAL (ng/ml)	Quartile 1 (0.0, 6.0)	Quartile 2 (6.0, 26.2)	Quartile 3 (26.2, 123.4)	Quartile 4 (123.4, 20245.2)	*P*-trend
Loop naïve, *n* (%)	3 (4%)	6 (7%)	6 (7%)	4 (5%)	.747
uNa concentration, mmol/l, mean (SD)	57.3 (27.9)	59.1 (27.6)	56.9 (30.2)	56.3 (27.7)	.704
FeNa, %, median [IQR]	2.4 [1.1, 3.9]	2.0 [0.8, 4.0]	1.9 [0.9, 4.0]	1.7 [0.7, 3.6]	.068
Total uNa over 72-h, mmol, mean (SD)^a^	547 (277)	559 (292)	466 (265)	512 (313)	.211
Total urine output over 72-h, ml, mean (SD)^a^	9234 (3078)	8969 (2847)	7704 (2377)	8288 (3342)	.012
Total loop diuretic dose before enrolment, mg, median [IQR]	80 [40, 130]	95 [40, 160]	80 [40, 120]	80 [50, 160]	.854
Total loop diuretic dose over 72-h, mg, median [IQR]^a^	600 [440, 900]	580 [350, 845]	520 [300, 760]	600 [400, 819]	.300
Thiazide used over 72-h, *n* (%)^a^	13 (19%)	19 (27%)	12 (19%)	15 (23%)	.912
Congestion score at enrolment, mean (SD)	9.1 (1.7)	9.6 (2.1)	9.1 (1.8)	9.2 (2.3)	.891
Congestion score at 24 h, mean (SD)^a^	5.4 (1.8)	6.2 (2.2)	6.0 (2.2)	6.3 (2.2)	.051
Congestion score at 48 h, mean (SD)^a^	4.4 (1.7)	5.2 (2.2)	5.5 (2.1)	5.6 (2.3)	.001
Congestion score at 72-h, mean (SD)^a^	3.9 (1.9)	4.5 (2.3)	4.5 (2.0)	4.7 (2.2)	.048

FeNa, fractional excretion of sodium; IQR, interquartile range; KIM-1, kidney injury molecule-1; mU/ml, milliUnits per millilitre; NAG, *N*-acetyl-β-D-glucosaminidase; NGAL, neutrophil gelatinase associated lipocalin; ng/ml, nanograms per millilitre; pg/ml, picograms per millilitre; SD, standard deviation; uNa, urine sodium.

^a^Only in 266 individuals with complete collections of 72-h urine.

### Association of kidney tubule biomarkers with baseline urinary sodium concentration and fractional excretion of sodium

**Figure 1:**
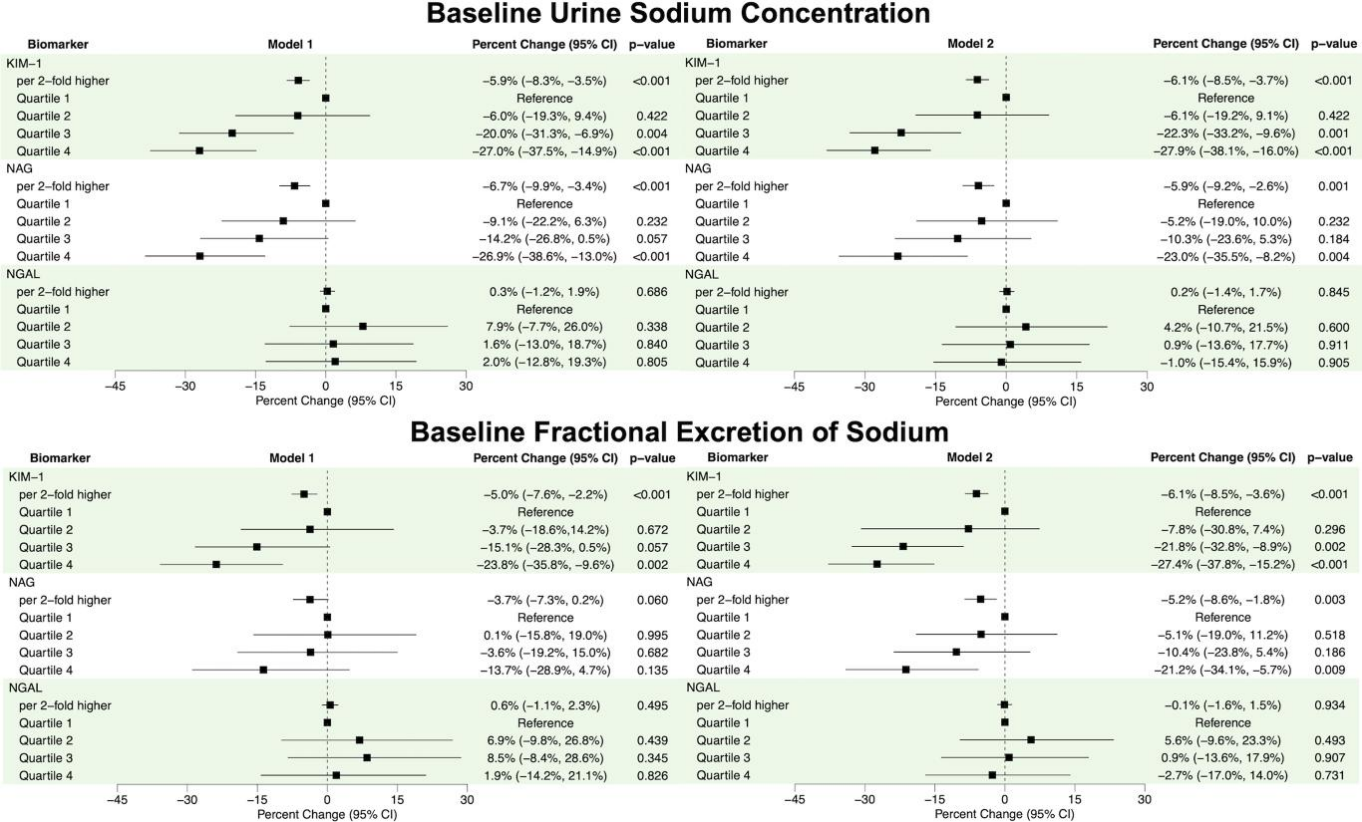
Forest plots of the associations of log2 and quartiles of urine tubule injury biomarkers with baseline urine sodium concentration and baseline fractional excretion of sodium at time of study enrolment in ROSE-AHF. Beta-coefficients reflect per cent change in urine sodium measurement per two-fold higher level of biomarker or relative to the first quartile. Model 1 adjusted for 1/urine creatinine. Model 2 adjusted for 1/urine creatinine, serum creatinine, blood urea nitrogen, hypertension, atrial fibrillation, diastolic blood pressure, outpatient diuretic dose, and N-terminal pro B-type natriuretic peptide levels. CI, confidence interval; KIM-1, kidney injury molecule-1; NAG, *N*-acetyl-β-D-glucosaminidase; NGAL, neutrophil gelatinase-associated lipocalin; NT-proBNP, N-terminal pro B-type natriuretic peptide; ROSE-AHF, Renal Optimization Strategies Evaluation Acute Heart Failure

**Figure 2:**
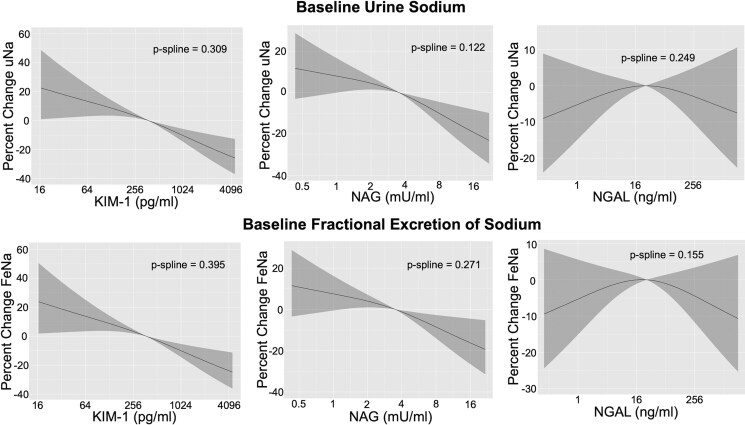
Spline plots of the associations of urine tubule injury biomarkers with baseline urine sodium concentration and baseline fractional excretion of sodium at time of study enrolment in ROSE-AHF. No significant non-linear associations were found. Model adjusted for 1/urine creatinine, serum creatinine, blood urea nitrogen, hypertension, atrial fibrillation, diastolic blood pressure, outpatient diuretic dose, and NT-proBNP levels. CI, confidence interval; KIM-1, kidney injury molecule-1; NAG, *N*-acetyl-β-D-glucosaminidase; NGAL, neutrophil gelatinase-associated lipocalin; NT-proBNP, N-terminal pro B-type natriuretic peptide; ROSE-AHF, Renal Optimization Strategies Evaluation Acute Heart Failure

The average uNa concentration at baseline was 57 ± 28 millimolar per litre (mmol/l). Higher values of KIM-1 and NAG were associated with lower uNa concentrations. Each two-fold increase in KIM-1 and NAG values was associated with 6.1% (3.7%, 8.5%) and 5.9% (2.6%, 9.2%) lower uNa concentration, respectively, in Model 2 (*Figure 1*). Urine sodium concentration decreased with increasing quartiles of KIM-1 and NAG, with those in the 4th quartile having 27.9% lower uNa concentration for KIM-1 and 23.0% lower for NAG, compared with those in the first quartile (*Figure 1*). Conversely, baseline urinary NGAL was not associated with uNa concentration (*Figure 1*). Spline analyses did not show significant non-linear associations for any biomarker with uNa (*Figure 2*). Associations did not vary by LVEF (*P*-interactions ≥.300) or eGFR categories (*P*-interactions ≥.095). When biomarkers were modelled together in Model 2, higher KIM-1 and NAG remained significantly associated with lower uNa, with a similar point estimate for KIM-1 but a lower estimate for NAG, while higher NGAL was associated with significantly higher uNa (*Table 3*). There was no indication of collinearity between biomarkers (*Table 3*).

**Table 3:** Association of biomarkers with baseline urine sodium measurements when modeled together

Biomarker (log2)	Urine sodium concentration			Fractional excretion of sodium		
	Per cent Change (95% CI)	*P*-value	VIF	Per cent Change (95% CI)	*P*-value	VIF
KIM-1	−6.3% (−8.8%, −3.6%)	<.001	1.34	−6.2% (−8.8%, −3.5%)	<.001	1.34
NAG	−4.0% (−7.4%, −0.5%)	.025	1.38	−3.2 (−6.9%, 0.3%)	.075	1.38
NGAL	1.7% (0.1%, 3.3%)	.036	1.28	1.4% (−0.2%, 3.1%)	.083	1.19

Model: 1/urine creatinine, serum creatinine, blood urea nitrogen, hypertension, atrial fibrillation, diastolic blood pressure, outpatient diuretic dose, and NT-proBNP levels.

CI, confidence interval; KIM-1, kidney injury molecule-1; NAG, NAG—*N*-acetyl-β-D-glucosaminidase; NGAL, neutrophil gelatinase-associated lipocalin; NT-proBNP, N-terminal pro B-type natriuretic peptide; VIF, variance inflation factor.

The median FeNa at baseline was 2.0% [0.8%, 4.0%]. Higher values of KIM-1 and NAG were associated with lower FeNa with each two-fold higher KIM-1 and NAG associated with 6.1% and 5.2% lower FeNa, respectively (*Figure 1*). FeNa decreased with increasing quartiles of KIM-1 and NAG with those in the 4th quartile having 27.4% lower FeNa for KIM-1 and 21.1% lower for NAG compared with those in the first quartile (*Figure 1*). Baseline urine NGAL was not associated with FeNa (*Figure 1*). Spline analyses did not show significant non-linear associations for any biomarker with FeNa (*Figure 2*). Associations did not vary by LVEF (*P*-interactions ≥.282) or eGFR categories (*P*-interactions ≥.121). When biomarkers were modelled together in Model 2, only higher KIM-1 was significantly associated with lower FeNa with no indication of collinearity between biomarkers (*Table 3*).

### Association of kidney tubule biomarkers with total urinary sodium output and urine volume over 72-h

The mean uNa excretion over the total of 72-h was 522 ± 288 mmol and mean urine volume was 8565 ± 2977 ml. Total uNa excretion was weakly correlated with baseline uNa concentration (*r* = 0.29, *P* < .001) and FeNa (*r* = 0.19, *P* = .002), but urine volume was not correlated with uNa concentration (*r* = 0.02 and *P* = .769) or FeNa (*r* = 0.07 and *P* = .246).

**Figure 3:**
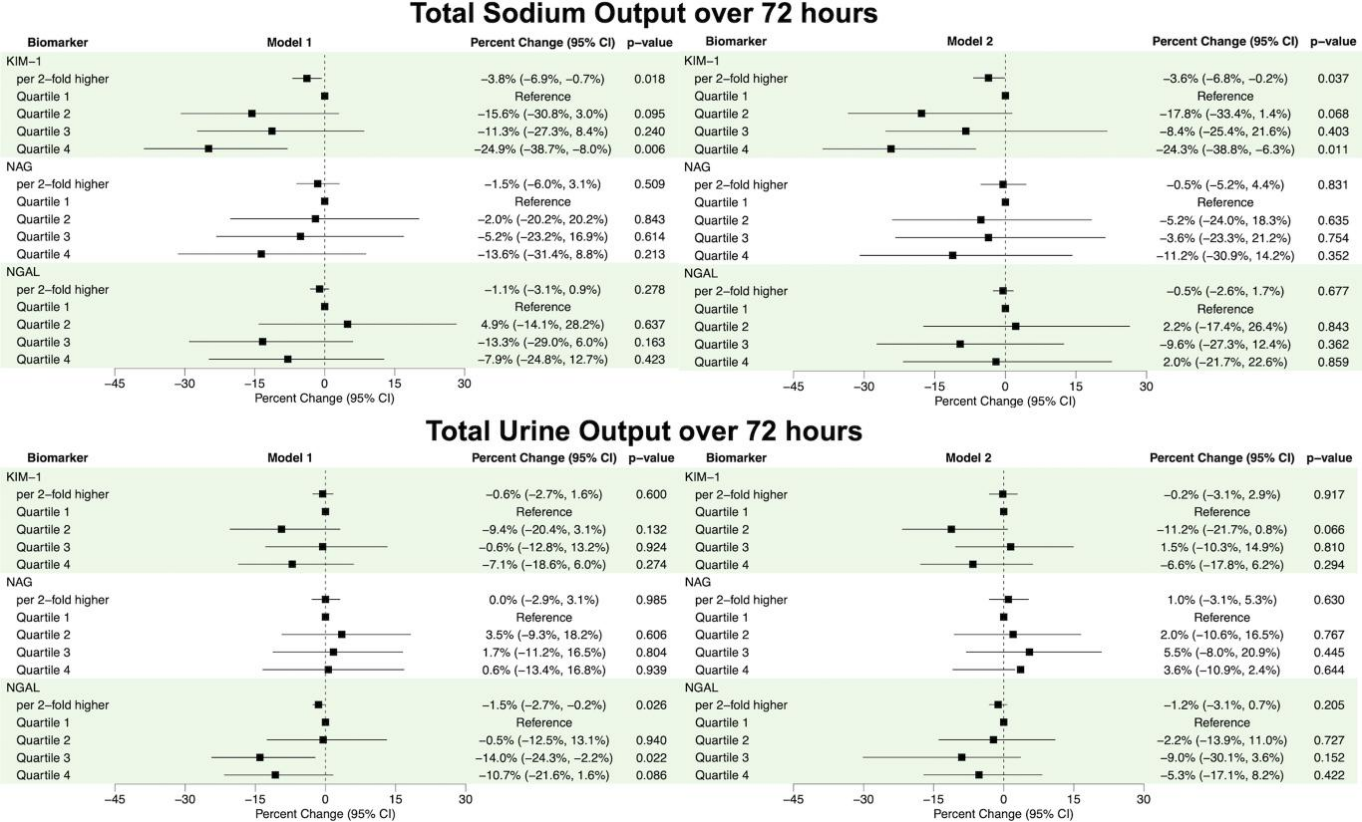
Forest plots of the associations of log2 and quartiles of urine tubule injury biomarkers with total urine sodium excretion and urine output at 72-h in ROSE-AHF. Beta-coefficients reflect per cent change in urine sodium measurement per two-fold higher level of biomarker or relative to the first quartile. Model 1 adjusted for 1/urine creatinine. Model 2 adjusted for 1/urine creatinine, serum creatinine, blood urea nitrogen, hypertension, atrial fibrillation, diastolic blood pressure, outpatient diuretic dose, N-terminal pro B-type natriuretic peptide levels, total loop diuretic dose, daily thiazide use, and daily congestion score. CI, confidence interval; KIM-1, kidney injury molecule-1; NAG, *N*-acetyl-β-D-glucosaminidase; NGAL, neutrophil gelatinase-associated lipocalin; NT-proBNP, N-terminal pro B-type natriuretic peptide; ROSE-AHF, Renal Optimization Strategies Evaluation Acute Heart Failure

**Figure 4:**
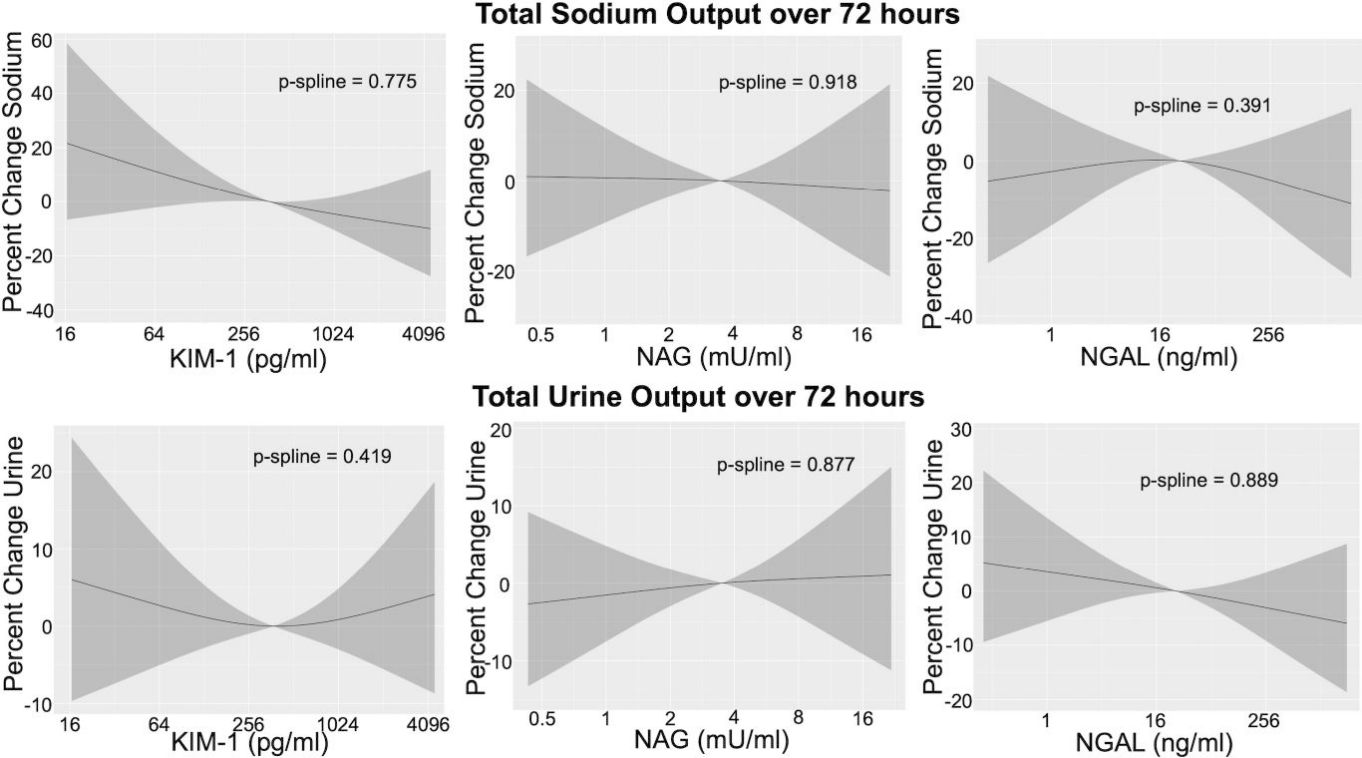
Spline plots of the associations of urine tubule injury biomarkers with total urine sodium excretion and urine output at 72-h in ROSE-AHF. No significant non-linear associations were found. Model adjusted for 1/urine creatinine, serum creatinine, blood urea nitrogen, hypertension, atrial fibrillation, diastolic blood pressure, outpatient diuretic dose, N-terminal pro B-type natriuretic peptide levels, total loop diuretic dose, daily thiazide use, and daily congestion score. CI, confidence interval; KIM-1, Kidney Injury Molecule-1; NAG, *N*-acetyl-β-D-glucosaminidase; NGAL, neutrophil gelatinase-associated lipocalin; NT-proBNP, N-terminal pro B-type natriuretic peptide; ROSE-AHF, Renal Optimization Strategies Evaluation Acute Heart Failure

Higher values of KIM-1 at baseline were associated with lower total uNa excretion over 72-h with 3.6% lower total uNa excretion per two-fold higher KIM-1 in Model 2 (*Figure 3*). Individuals in the fourth quartile had 24.3% lower uNa excretion compared with the first quartile (*Figure 3*). Neither NAG nor NGAL were significantly associated with total uNa excretion over 72-h (*Table 4*). Spline analyses did not show significant non-linear associations for any biomarker with total uNa excretion (*Figure 4*).

**Table 4:** Association of biomarkers with total urine sodium excretion and urine output at 72 h when modeled together

Biomarker (log2)	Total urine sodium extretion			Total urine output		
	Per cent Change (95% CI)	*P*-value	VIF	Per cent Change (95% CI)	*P*-value	VIF
KIM-1	−3.9% (−7.4%, −0.2%)	.039	1.35	0.2% (−3.0%, 3.5%)	.915	1.35
NAG	1.0% (−3.9%, 6.1%)	.701	1.44	1.2% (−3.1%, 5.7%)	.593	1.44
NGAL	0.2% (−3.1%, 2.5%)	.865	1.23	−1.3% (−3.2%, 0.7%)	.198	1.23

Model: 1/urine creatinine, serum creatinine, blood urea nitrogen, hypertension, atrial fibrillation, diastolic blood pressure, outpatient diuretic dose, and NT-proBNP levels, total diuretic dose, daily thiazide use, daily congestion score.

CI, confidence interval; KIM-1, kidney injury molecule-1; NAG, NAG—*N*-acetyl-β-D-glucosaminidase; NGAL, neutrophil gelatinase-associated lipocalin; NT-proBNP, N-terminal pro B-type natriuretic peptide; VIF, variance inflation factor.

Associations did not vary by treatment arm (*P*-interaction ≥.057). Associations did not vary by LVEF for KIM-1 and NAG (*P*-interactions ≥.192), but were significantly different for NGAL (*P*-interaction = .028) such that higher NGAL was directionally associated with lower uNa excretion in individuals with LVEF <50% (−1.9% per two-fold higher NGAL, 95% CI −4.3%, 0.6%, *P*-value = .142) but directionally associated with higher uNa in individuals with LVEF ≥50% (3.8% per two-fold higher NGAL, 95% CI −0.6%, 8.4%, *P*-value = .093). Associations did not vary by eGFR categories for KIM-1 and NAG (*P*-interactions ≥.075), but were significantly different for NGAL and the eGFR categories of ≥45 ml/min/1.73 m^2^ and 30–<45 ml/min/1.73 m^2^ (*P*-interaction = .024) such that higher NGAL was directionally associated with higher uNa excretion in individuals with eGFR ≥45 ml/min/1.73 m^2^ (3.3% per two-fold higher NGAL, 95% CI −0.6%, 7.5%, *P*-value = .101) but directionally associated with lower uNa in individuals with eGFR 30–<45 ml/min/1.73 m^2^ (−2.5% per two-fold higher NGAL, 95% CI −5.8%, 0.9%, *P*-value = .142). Interactions were not significant between NGAL and other eGFR categories though (*P*-interactions ≥.110). When biomarkers were modelled together in Model 2, only higher KIM-1 was significantly associated with lower uNa excretion with no indication of collinearity between biomarkers (*Table 4*).

Regarding total urine output over 72-h, only higher values of NGAL at baseline were associated with lower urine output with each two-fold increase in NGAL associated with 1.5% lower urine output in model 1, but this association was no longer significant after adjusting for confounders (*Figure 3*). Spline analyses did not show significant non-linear associations for any biomarker with total urine output (*Figure 4*). Associations did not vary by treatment arm (*P*-interaction ≥.241), LVEF (*P*-interactions ≥.084), or eGFR categories for NAG and NGAL (*P*-interactions ≥.065); however, they did vary by eGFR categories for KIM-1. There was a significant interaction between the eGFR categories of ≥45 ml/min/1.73 m^2^ and 30–<45 ml/min/1.73 m^2^ (*P*-interaction = .017) such that higher KIM-1 was associated with higher urine output in individuals with eGFR ≥45 ml/min/1.73 m^2^ (3.3% per two-fold higher KIM-1, 95% CI 0.1%, 6.5%, *P*-value = .045) but directionally associated with lower urine output in individuals with eGFR 30–<45 ml/min/1.73 m^2^ (−1.9% per two-fold higher KIM-1, 95% CI −4.8%, 1.0%, *P*-value = .200). Interactions were not significant between KIM-1 and other eGFR categories (*P*-interactions ≥.214). When biomarkers were modelled together in Model 2, all remained unassociated with total urine output (*Table 4*).

## Discussion

In this study of patients hospitalized with AHF and kidney dysfunction, greater tubule injury was associated with lower uNa concentration and FeNa at baseline and lower total uNa output over 72-h. This association was most consistently seen for KIM-1 across all measures of uNa, while only higher NAG at baseline was associated with lower uNa concentrations and FeNa at baseline. Conversely, none of the biomarkers were associated with urine output when accounting for confounders. These findings suggest that tubular injury may contribute to greater sodium avidity and impaired diuretic responsiveness in patients with AHF.

Kidney function in HF has traditionally been assessed by eGFR, which is an important indicator of the kidney’s ability to filter and deliver sodium to the tubular system.^23^ However, uNa excretion is primarily regulated by the handling of different segments of the kidney tubule, and the proximal tubule is responsible for the largest percentage of sodium reabsorption. Additionally, loop diuretics need to be secreted by the proximal tubule to be delivered to their site of action on the luminal surface of the thick ascending limb of the loop of Henle. Therefore, dysfunction of the proximal tubule may lead to reduced loop diuretic secretion or enhanced sodium reabsorption.^7^ While the biomarkers evaluated here primarily reflect proximal tubule injury, the proximal tubules are near the distal tubules, so it remains possible that global dysfunction of tubules may impair distal tubule sodium handling or reduce the action of loop diuretics in the thick ascending limb.^8,24^ These factors impairing sodium handling could contribute to persistent congestion and worse outcomes in AHF.

Our hypothesis about the proximal tubule’s role in sodium avidity is supported in part by the specific sites of production of the kidney tubule injury markers studied and by the strength of the associations observed in our analysis. Kidney injury molecule-1 is a marker of kidney tubule injury that is specific to the kidney and only produced in the proximal tubule.^25,26^ During ischaemic, toxic, or haemodynamic stress, proximal tubular epithelial cells rapidly upregulate membrane KIM-1, leading to an early rise in urinary KIM-1. *N*-acetyl-ß-D-glucosaminidase is also a proximal tubule specific biomarker released from lysosomes upon injury, indicating proximal tubular injury and dysfunction.^27^ Because NAG is a large lysosomal hydrolase that is not filtered at the glomerulus, detectable urinary NAG primarily reflects release from damaged proximal tubular cells. Conversely, NGAL has been associated with injury in both the proximal and distal tubules and can also be derived from circulating neutrophils. Thus, urinary NGAL rises when distal tubular production increases or when proximal tubular reabsorption is impaired, making it a broader but less proximal-specific signal of injury.^28^ In the current analysis, KIM-1 and NAG were associated with lower natriuresis, whereas NGAL was not. Given that ∼65% to 75% of sodium is reabsorbed in the proximal tubule, while the loop of Henle and distal tubule account for only 25%–35%, injury to the proximal tubule is more likely to disrupt sodium handling.^29^ These distinct sites of biomarker expression, together with the segment-specific differences in sodium reabsorption, likely explain why elevations in urinary biomarkers specific to the proximal tubule were associated with greater sodium avidity. Furthermore, previous studies have suggested that different segments of the kidney tubules may have varying susceptibility to acute congestion-related damage. A study of 30 patients with chronic HF investigated the effects of loop diuretic withdrawal on tubular injury biomarkers.^30^ Participants discontinued loop diuretics for 72-h, after which their diuretic regimen was reinstated. During diuretic withdrawal, there was a significant rise in urinary KIM-1 and urinary NAG, while urinary NGAL remained unchanged. These findings suggest that the proximal tubules, compared with the distal tubules, are more vulnerable to congestion-related renal impairment. Elevations in these injury markers may indicate acute tubular injury, greater sodium avidity, and reduced diuretic response, even before distal tubular injury markers become elevated.

The ROSE-AHF trial enrolled patients with AHF across the full spectrum of LVEF. Although we did not observe a consistent overall interaction between LVEF and the associations of urinary biomarkers with natriuresis, stratified analyses suggested that higher NGAL was associated with lower uNa excretion in participants with LVEF <50%, but with higher uNa excretion in those with LVEF ≥50%. Because NGAL is also released from neutrophils and serves as a systemic inflammation and fibrosis marker, higher NGAL levels may reflect greater HF severity rather than isolated tubular injury.^28^ Thus, in patients with AHF and LVEF <50%, greater urinary NGAL and sodium avidity may both be markers of more advanced disease rather than being directly linked to each other. However, the mechanism underlying the opposite association in those with LVEF ≥50% is unclear, and the possibility of a type I error should be considered. Thus, these findings should be interpreted as hypothesis generating.

We also evaluated whether baseline kidney function modified associations between tubular biomarkers and measures of decongestion. We observed statistically significant interactions between eGFR and NGAL for 72-h total uNa excretion and between eGFR and KIM-1 for 72-h urine output. These interactions were limited to comparisons between individuals with eGFR ≥45 and 30–<45 ml/min/1.73 m^2^ and were not observed for other eGFR strata. Importantly, no consistent interaction pattern was seen across biomarkers, outcomes, or eGFR categories, and interactions involving the lowest eGFR category (<30 ml/min/1.73 m^2^) were not statistically significant. The lack of consistency across strata may reflect limited statistical power, as fewer participants had available 72-h measures and the smallest proportion had eGFR <30 ml/min/1.73 m^2^. Alternatively, these findings may represent chance associations arising from multiple interaction tests. Accordingly, these findings should be interpreted cautiously and require further study, but the greater consistency of tubular biomarker associations in participants with lower eGFR is of potential significant clinical value as diuretic response can be variable in patients with kidney disease.

Our study has multiple strengths, including a well-characterized study population from a multicentre study, high-quality data on uNa excretion at multiple endpoints, as they were the parent trial’s primary endpoint, and measurement of multiple biomarkers, all measured in a core lab. Our study also has important limitations. First, the sample size was relatively small, limiting our ability to evaluate hard endpoints such as death and HF hospitalization. The small sample size, especially in each treatment arm, may have impacted our ability to identify other differences in biomarker performance based on treatment. Second, as a retrospective analysis, residual confounding cannot be fully eliminated despite multivariable adjustments. Third, as a cross-sectional study, our findings are only hypothesis generating and we cannot prove causality between tubular injury and either enhanced sodium avidity or impaired diuretic responsiveness. Fourth, the external validity of these findings is limited by the characteristics of the ROSE-AHF population, which included individuals with reduced kidney function (eGFR 15–60 ml/min/1.73 m^2^), predominantly older men, and a high prevalence of ischaemic cardiomyopathy. Fifth, the cross-sectional nature of the natriuresis and biomarker measurements raises the possibility that low uNa may reflect heightened neurohormonal activation that simultaneously contributes to tubular stress and biomarker elevation, rather than impaired natriuresis resulting from tubular injury. Sixth, urinary biomarkers were assessed only at baseline and temporal trajectories might better distinguish transient stress from sustained injury. Seventh, this analysis did not include post-discharge clinical outcomes, limiting the ability to determine how biomarker–natriuresis relationships translate into longer-term prognosis. Lastly, the use of additional diuretic agents, such as acetazolamide, was not recorded and may have resulted in residual confounding.

In conclusion, higher values of tubular injury biomarkers in patients with AHF are associated with greater sodium avidity and impaired diuretic responsiveness. These findings suggest that tubular injury may be an under-recognized contributor to sodium retention and diuretic resistance in HF.

## Supplementary Material

xvag079_Supplementary_Data

## Data Availability

No data were generated or analysed for this manuscript.

## Pre-registered Clinical Trial Number

None supplied.
