# Supplementary material for: Kidney tubule injury is associated with sodium avidity and diuretic responsiveness in acute heart failure
Source: ESC Heart Fail. 2026 Mar 13;13(2):xvag079. doi: 10.1093/eschf/xvag079 (PMC13037371; doi:10.1093/eschf/xvag079)
Supplement: xvag079_Supplementary_Data [file xvag079_supplementary_data.docx]

**Supplemental Material**

**Supplemental Figure S1. Heatmap of the correlation between markers of glomerular filtration function and markers of tubular injury**

Correlations were evaluated using Spearman rank-order correlation analysis.

eGFR – estimated Glomerular Filtration Rate; KIM-1 - Kidney Injury Molecule-1; NGAL - Neutrophil Gelatinase-Associated Lipocalin; NAG - N-acetyl-β-D-glucosamine

| **Supplemental Table 1. Comparison of individuals with and without urine sodium data at 72-hours** | | | |
| --- | --- | --- | --- |
|  | Excluded | Included | p-value |
|  | n = 73 | n = 266 |  |
| Age, years, mean (SD) | 68 (12) | 70 (12) | 0.229 |
| Male, n (%) | 53 (73%) | 195 (73%) | 1.000 |
| Race, n (%) |  |  | 0.164 |
| White | 51 (70%) | 204 (77%) |  |
| Black | 21 (28%) | 49 (18%) |  |
| Other | 1 (1%) | 11 (4%) |  |
| Unknown | 0 (0%) | 2 (1%) |  |
| BMI, kg/m^2^, mean (SD) | 32.8 (8.3) | 32.3 (7.9) | 0.638 |
| Years of heart failure, mean (SD) | 6.3 (5.8) | 6.2 (6.1) | 0.917 |
| LVEF, %, mean (SD) | 36 (17) | 37 (17) | 0.769 |
| Systolic blood pressure, mmHg, mean (SD) | 120 (20) | 117 (18) | 0.185 |
| Diastolic blood pressure, mmHg, mean (SD) | 67 (13) | 66 (11) | 0.487 |
| Ischemic cardiomyopathy, n (%) | 40 (55%) | 157 (59%) | 0.607 |
| History of myocardial infarction, n (%) | 27 (37%) | 93 (35%) | 0.855 |
| Hypertension, n (%) | 62 (85%) | 221 (83%) | 0.842 |
| Atrial fibrillation, n (%) | 45 (62%) | 159 (60%) | 0.878 |
| COPD, n (%) | 27 (37%) | 61 (23%) | 0.023 |
| Diabetes, n (%) | 37 (51%) | 152 (57%) | 0.395 |
| Hyperlipidemia, n (%) | 58 (80%) | 208 (78%) | 0.944 |
| ACEi or ARB, n (%) | 33 (45%) | 133 (50%) | 0.553 |
| Beta-blocker, n (%) | 65 (89%) | 217 (82%) | 0.182 |
| MRA, n (%) | 28 (38%) | 71 (27%) | 0.072 |
| Outpatient loop dose, mg, median [IQR] | 80 [40, 240] | 80 [40, 160] | 0.602 |
| BUN, mg/dL, mean (SD) | 39 (19) | 43 (22) | 0.13 |
| Creatinine, mg/dL, mean (SD) | 1.67 (0.53) | 1.75 (0.55) | 0.279 |
| Cystatin C, mg/dL, mean (SD) | 1.77 (0.58) | 1.83 (0.56) | 0.385 |
| eGFR, ml/min/1.73 m^2^, mean (SD) | 45 (13) | 42 (14) | 0.186 |
| eGFR Categories |  |  | 0.395 |
| eGFR <30 ml/min/1.73 m^2^, n (%) | 11 (15%) | 58 (22%) |  |
| eGFR 30 -<45 ml/min/1.73 m^2^, n (%) | 27 (37%) | 98 (37%) |  |
| eGFR ≥45 ml/min/1.73 m^2^, n (%) | 35 (48%) | 110 (41%) |  |
| NT-proBNP, pg/ml, median [IQR] | 3961 [2344, 8620] | 5272 [2308, 10331] | 0.216 |
| KIM-1, pg/ml, median [IQR] | 38.1 [7.3, 144.8] | 24 [6.0, 120.7] | 0.457 |
| NAG, mU/ml, median [IQR] | 3.51 [1.98, 6.14] | 3.48 [1.68, 6.22] | 0.920 |
| NGAL, ng/ml, median [IQR]) | 308.5 [154.3, 719.6] | 400.6 [141.7, 1011.7] | 0.253 |
| UACR, mg/g, median [IQR] | 23 [6, 78] | 14 [4, 65] | 0.222 |
| Urine sodium, mmol/L, mean (SD) | 57.1 (26.2) | 57.5 (28.8) | 0.926 |
| FeNa, %, median [IQR] | 1.8 [0.9, 3.2] | 2.1 [0.8, 4.1] | 0.232 |
| Treatment arm |  |  | 0.134 |
| Placebo, n (%) | 17 (23%) | 95 (36%) |  |
| Dopamine, n (%) | 29 (40%) | 87 (33%) |  |
| Nesiritide, n (%) | 27 (37%) | 84 (32%) |  |
| ACEi - Angiotensin-Converting Enzyme Inhibitor; ARB - Angiotensin Receptor Blocker; AF - Atrial Fibrillation; BMI - Body Mass Index; BUN - Blood Urea Nitrogen; COPD - Chronic Obstructive Pulmonary Disease; eGFR – estimated glomerular filtration rate; KIM-1 - Kidney Injury Molecule-1; LVEF - Left Ventricular Ejection Fraction; MRA - Mineralocorticoid Receptor Antagonist; NGAL - Neutrophil Gelatinase-Associated Lipocalin; NAG - N-acetyl-β-D-glucosaminidase; NT-proBNP - N-terminal pro B-type Natriuretic Peptide; UACR - Urine Albumin-to-Creatinine Ratio | | | |

| **Supplemental Table 2: Components and scores of the composite congestion score.** | |
| --- | --- |
| Component | Points |
| Jugular Venous Pressure |  |
| <8 cm | 0 |
| 8-12 cm | 1 |
| 13-16 cm | 2 |
| >16 cm | 3 |
| Peripheral Edema |  |
| None | 0 |
| 1+ | 1 |
| 2+ | 2 |
| 3+ | 3 |
| 4+ | 4 |
| Orthopnea |  |
| No pillows | 0 |
| One pillow (10 cm) | 1 |
| Two pillows (20 cm) | 2 |
| Three or more pillows | 3 |
